# How loan descriptions affect the likelihood that borrowers obtain loans in P2P networks? -An empirical analysis based on the "Renrendai" platform

**DOI:** 10.1371/journal.pone.0283508

**Published:** 2023-09-07

**Authors:** Qiao Sun, Jigan Wang, Hao Zhang, Ting Wen

**Affiliations:** 1 Business School, Hohai University, Nanjing, Jiangsu Province, China; 2 School of Economics and Management, Jiangsu University of Science and Technology, Zhenjiang, Jiangsu Province, China; 3 School of Business Administration, Nanjing University of Finance & Economics, Nanjing, Jiangsu Province, China; BeiHang University School of Economics and Management, CHINA

## Abstract

Information asymmetry is widespread in the P2P online lending market, creating an imbalance in the position of lenders and borrowers. This paper aims to expand the process of information exchange between lenders and borrowers by analyzing the link between soft information such as borrowers’ loan descriptions and lending outcomes. Based on the transaction data of the ‘Renrendai’ platform, this paper analyzed the linguistic features and extracted the content of loan descriptions using a latent Dirichlet allocation (LDA) theme model. To further explore the value of loan descriptions in predicting lending success, this paper conducts a prediction study based on a support vector machine model. It is found that: lenders focus on effective information in the loan descriptions, the linguistic complexity affects the transaction, with simple and direct statements being more favorable; the content for building a good personal image of the borrower will significantly contribute to the lending success. In the prediction study section, it is demonstrated that loan descriptions’ language feature indicators can improve prediction accuracy. This paper uncovers the importance of loan descriptions in online lending transactions, which has implications in assisting lenders’ investment judgments, as well as in platform information system improvements.

## 1 Introduction

P2P network lending is a model that emerged along with the innovation of Internet finance, which is characterized by a low entry threshold, no mortgage, convenience, etc. [1]. China has become the largest P2P lending market in the world. At the same time, the trust problem between the borrower and lender increases the credit risk. P2P platforms mainly act as information intermediaries, and many of them try to solve the problem of mistrust by providing guarantees and other means. However, lenders are generally in a predicament of information weakness. The platform cannot accurately verify the authenticity of borrowers’ personal information. Moreover, China lacks a matching personal credit reference system. Although some platforms have tried to solve this problem by establishing user databases, building analysis models, and other means, the results are still very limited.

The transaction process of online lending avoids the involvement of financial institutions. and accompanied by a serious information asymmetry problem [2]. Information asymmetry may lead to two behavioral tendencies: adverse selection and moral hazard [3]. In the process of online lending, investors are at an obvious information disadvantage [4], and can only make loan decisions based on borrowers’ personal information provided by the platform [5]. The information of borrowers that can be accessed is provided by the online lending platform. Studies have proven that credit rating systems can alleviate the problem of information asymmetry. However, a large proportion of P2P platform users are capital demanders who have difficulty obtaining loans from banks and other institutions, they are more likely to give false information [6].

Borrowers’ information can generally be divided into hard and soft information. Hard information refers to information that can be verified, and soft information refers to information that cannot be directly verified. Such information mainly includes quantified information such as age, gender income level, in addition to text-based information such as loan descriptions. Compared with hard information, soft information is more abundant. In P2P lending, the borrower will provide the loan description text related to his/her situation and loan status, which belongs to the information voluntarily disclosed by him/her. Subjective information often includes some information that the borrower does not intend to provide, and the text characteristics and organizational pattern of the text will carry effective information related to the borrower [7]. A loan description is an original information about borrowers that lenders can access, for platforms generally do not process the text content. In addition, when the borrower writes text information, it also includes a great deal of information voluntarily disclosed. Some indirect information contained in the text can be used on a supplementary basis for investors to measure risks [8]. The loan description text can be quantified through natural processing and other means to extract and analyze the semantic features and content, thus providing a basis for analyzing the credit risk of network lending [9]. The research on text description has generally focused on marketing and other fields and has found that the convenience of text reading will significantly affect product sales and company performance [10, 11].

To analyze the impact of loan descriptions on lending outcomes and to explore the potential of descriptions for improvement in outcome prediction accuracy, this paper conducted a regression analysis and prediction study. This paper contributes as follows: (1) using text processing methods and the LDA model, evaluation indicators reflecting the text and content characteristics of loan descriptions were constructed; (2) a regression model between linguistic features of loan descriptions and loan success was built and found that a negative correlation between linguistic complexity and loan success and personal image-building descriptions influence investors’ judgment; (3) using SVM model, it was confirmed that loan descriptions can significantly improve accuracy in predicting loan outcomes.

The remainder of the paper is arranged as follows: Section 2 is the method framework used in this paper, including data resources, the quantification of the loan description text, and the construction of related indicators, as well as the construction of the regression model. The empirical analysis is conducted in Section 3. Finally, we discuss the results of the paper and propose some suggestions in Section 4.

## 2 Methodology

### 2.1 Data

#### 2.1.1 Data source

The sample data in this paper are from “Renrendai”, one of the P2P online lending platforms in China Founded in 2010(Renrendai is one of the earliest online lending information intermediary services in China. It is committed to providing professional personal financial information and loan-matching services for the high-growth population. Up to 2020, the accumulated transaction amount has exceeded 117 billion CNY).

#### 2.1.2 Data content and non-privacy statement

We randomly recorded the loan records between transaction order numbers 395174 and 794592. The status of platform lending transactions is divided into three types: successful, failed, and in progress. In this paper, the data collection completion time is used as the cutoff, and the completed transaction data are selected for compilation. The completion of the loan order is divided into two categories: successful loans and failed loans. After the data cleansing process, 20,349 loan data points remain. Among them, the number of orders successfully obtained through borrowing was 4,197, while the number of failed loans was 16,152.

Several types of information are included in the data used in this study; lending order information, borrower information that is mandatorily disclosed by the platform, borrower credit history, and voluntarily disclosed information. Loan orders information includes amount, interest rate, etc. Borrower’s public information includes job, income, etc. Credit information includes credit certification and historical number of borrowings, etc. Voluntary disclosures such as loan description, which include additional information about the borrower’s origination of this loan, including the purpose of the loan, personal income level, etc.

The data collection process is carried out in compliance with the requirements of the Renrendai website. It was obtained by collating the publicly available loan orders after completing the registration as an investor on that website. In addition, the data in this study does not involve private information about the borrowers, and the data that were processed, especially the loan descriptions, were also anonymized and desensitized.

### 2.2 Quantification of loan description and variable design

#### 2.2.1 Loan description linguistic feature quantification

The length of the loan description reflects the importance attached to it by the borrower, and too short a description may make investors think that the borrower lacks confidence in his or her repayment ability [12]. The amount of information contained in the text is also related to the length of the text. The longer the description is, the more information the borrower voluntarily discloses, and the more in-depth understanding investors have, so they can make investment decisions. Borrowers who lack hard information, are more inclined to provide detailed descriptions to obtain loans.

For readers, easy-to-read texts are easier to understand and more impressive than unfamiliar descriptive texts. Texts with high complexity tend to bring about disturbing information, which increases the difficulty of readers in processing information [13]. By constructing a complexity index, Chen [14] studied the readability of loan descriptions and found that the less complex the description text is, the more positive the signal it can provide to investors, thus significantly improving the success rate of loans. The more clauses there are, the more complex the composition of the loan description will be, and the number of clauses can be reflected by the number of periods. Moreover, Ye [15] found that the use of punctuation has a significant impact on the success rate, which can play the role of psychological incremental information.

In this paper, Python 3.0 and Chinese text processing software (Wenxin) were used to carry out a series of operations, such as Chinese word segmentation on loan descriptions. The ratio of complex words to all words in each description text was calculated. We also collated the number of periods to measure the total number of clauses that made up the loan description text to reflect the complexity of the text structure composition. The validity of the descriptive text was demonstrated by calculating the total length of the text and the proportion of valid content. **Table 1** presents a detailed description of each linguistic feature variable.

**Table 1:** Linguistic features variables of the loan description.

Variable name	Instruction
Length of sentence (length)	The total number of characters in a single description text; Chinese characters account for 2 characters, and punctuation marks, spaces and numbers account for 1 character each.
Valid characters	The number of characters in a text after removing invalid content such as spaces and punctuation.
Ratio of effective characters	Valid characters/length of sentence
Periods	The total number of periods in the loan description text
Ratio of complex words	Words with more than four characters are denoted as complex words: the ratio of the characters of complex words in a single text to the total number of characters

### 2.2.2 Loan description content feature extraction

The loan description contains some specific content and feature descriptions, which can mitigate the impact of information asymmetry on lenders. Therefore, the description of content features has become one of the main bases for investors to make decisions [16]. Dorfleitner [17] found that the characteristic information indicating the borrower’s social and emotional connotation could improve his/her probability of success by manually marking keywords in the loan description in the German language environment. Herzenstein [18] summarized 1,493 loan application samples from the "Prosper" platform and found six language dimensions of text content, namely, "reliability", "success", "economic hardship", "religious belief", "moral integrity" and "diligence".

In P2P online lending, the loan description, as a part of the borrowers’ subjective provision of information, may contain some unique personal information and important information that can attract investors to make decisions [19]. At the same time, the borrower will create a good image, which will increase the trust of lenders. The increase in the trust will greatly increase the probability of loan success. Of course, the language is also true or false. Borrowers can help lenders have a more comprehensive understanding of themselves through self-statements, or they can disguise themselves through language, which may mislead lenders.

The loan is described as text information. To analyze the specific content more intuitively, it is necessary to extract and quantify the characteristic information. Therefore, this paper adopts the mature LDA subject extraction model in natural language processing (NLP) to analyze the loan description [20].

**Fig 1:**
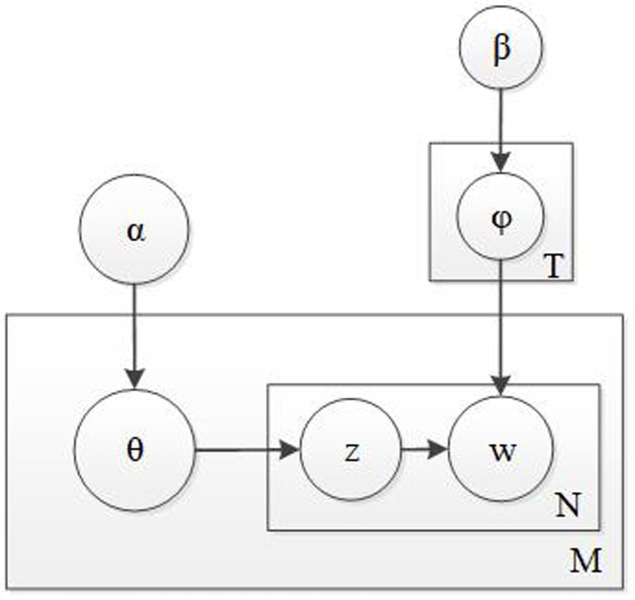
The structure of the LDA theme extraction model.

The LDA (latent Dirichlet allocation) model is a three-layer Bayesian probabilistic model, which can be divided into three layers: documents, themes, and words. The model structure is shown in **Fig 1**. The LDA model holds that a document consists of multiple themes, with each theme corresponding to different words, which is a polynomial distribution. The document to the theme is subject to a Dirichlet distribution.

In **Fig 1**, the probabilistic distribution of the target document to the hidden subject is “θ”, obeying the Dirichlet distribution with parameter “α”. “φ” represents the probabilistic distribution of the word corresponding to the subject, obeying the Dirichlet distribution with parameter “β”. “T” represents the number of themes, “N” is the number of words in a document, and “M” is the number of documents in the document collection. “z” indicates the number of hidden themes for each word in the document, and “w” represents the word vector of the target document.

In this paper, the LDA toolkit is used to extract the theme of loan description content. According to the distribution of subject words, four subjects with distinct meanings were selected from the extracted subjects as the content features of loan description, namely, “working condition” (Theme 1), “loan purpose” (Theme 2), “asset” (Theme 3) and “image-building content” (Theme 4). Among them, image-building content refers to the content about credit written by the borrower, which is conducive to building a good image of himself/herself to improve his/her possibility of obtaining loans. **Table 2** below shows the distribution of some of the subject words of each content theme.

**Table 2:** The distribution of subject words.

Variable	Theme	Example of subject words
Theme 1	Working condition	职员(clerk) 法人(legal person) 职工(employee) 公司(corporation) 个体(self-employed) 零售业(retail industry) 制造业(manufacturing industry) 公务员(civil servant)
Theme 2	Loan purpose	周转(turnover of funds) 经营(business) 供应(supply) 装修(decoration) 租赁(lease) 购房(house-purchase) 扩大(expand) 生产(production)
Theme 3	Asset	认证(certification) 审核(check) 房屋(house) 房子(house) 收入(income) 工资(wage) 现居(current location)万元(ten thousand CNY)
Theme 4	Image-building content	支持(support) 希望(hope) 谢谢(gratitude) 真实(real) 标准(standard) 提供(provide) 稳定(stable) 信用(credit)

### 2.3 The construction of regression models

For studies on P2P online lending, variables are usually selected from demographic characteristics, social assets, credit status, and other aspects. To analyze the influence of loan description information on loan acquisition, it is necessary to establish the correlation variable of hard information as the control variable before regression analysis. Based on control variables, the relevant variables of the loan description information were added to the regression model to analyze the degree of influence on the dependent variable (loan acquisition).

#### 2.3.1 Establishment of control variables

Based on previous studies [21, 22] and combined with sample data, this paper selects a total of 5 variables as the initial model controls for empirical analysis from the above aspects. The control variables are the amount of the loan (log), the educational level of the borrower, the credit limit (log), the credit report, and the number of successful borrowings. The initial regression model (1) was established. The definition and assignment of control variables are shown in **Table 3**.


$$Logistic(Success)=\alpha_{i}\;*\;Controls\;+\;\varepsilon_{i}$$ (1)

**Table 3:** Interpretation and assignment of control variables.

	Variable name	Interpretation and assignment
Dependent variable (success)	Access to loans or not (success)	If the bid is full within the specified time, then the weight is 1, and otherwise, the weight is 0
Control variable (controls)	Amount of the loan (amount)	Order borrowing amount, logarithmic processing
	Educational level	Borrower’s educational level, with a total of four options: high school or below, junior college, undergraduate, graduate or above; the values are 1, 2, 3, and 4, respectively
	Credit limit	The maximum amount of the borrower’s loan on the platform is processed logarithmically
	Credit report	Does the borrower provide a credit report? Yes, 1, no, 0
	Successful record	The number of times the borrower has successfully borrowed money on the credit platform

#### 2.3.2 The regression model with loan description variables

*(1) Linguistic variables*. A text analysis of the loan description is carried out. According to the analysis results, the linguistic variables (linguistic variables) are introduced, and the regression model (2) is established.


$$Logistic(Success)=\alpha_{i}\;*\;Controls\;+\;\beta_{i}*\;LinguisticVariables\;+\;\varepsilon_{i}$$ (2)

The validity of the loan description expression can be reflected by the length of the text and the proportion of valid characters. Chinese word segmentation software was used to calculate the ratio of complex words and the number of clauses according to the number of periods to reflect the complexity of the loan description.

*(2) Content variables*. The loan is described as text information. To analyze the specific content more intuitively, it is necessary to extract and quantify the characteristic information. To this end, this paper adopts the LDA theme extraction model to analyze the loan description and adds the theme extraction results (theme variables) to the regression model to establish model (3).


$$Logistic(Success\;)=\alpha_{i}\;*\;Controls\;++\;\gamma_{i}\;*\;TopicVirables\;+\;\varepsilon_{i}$$ (3)

### 2.4 The SVM model for the prediction of lending results

There are only two statuses for the loan outcome. One is that the loan order is completed within the specified time and the loan amount is successfully obtained. Second, the loan order fails to reach the required total bidding amount within the specified time, and the loan fails. So the prediction research on online lending results is a dichotomy problem [23]. In the prediction research of the P2P lending market, the existence of soft information variables leads to nonlinear problems, while machine learning and classification models can be well applied to the prediction research of network lending data [24].

**Fig 2:**
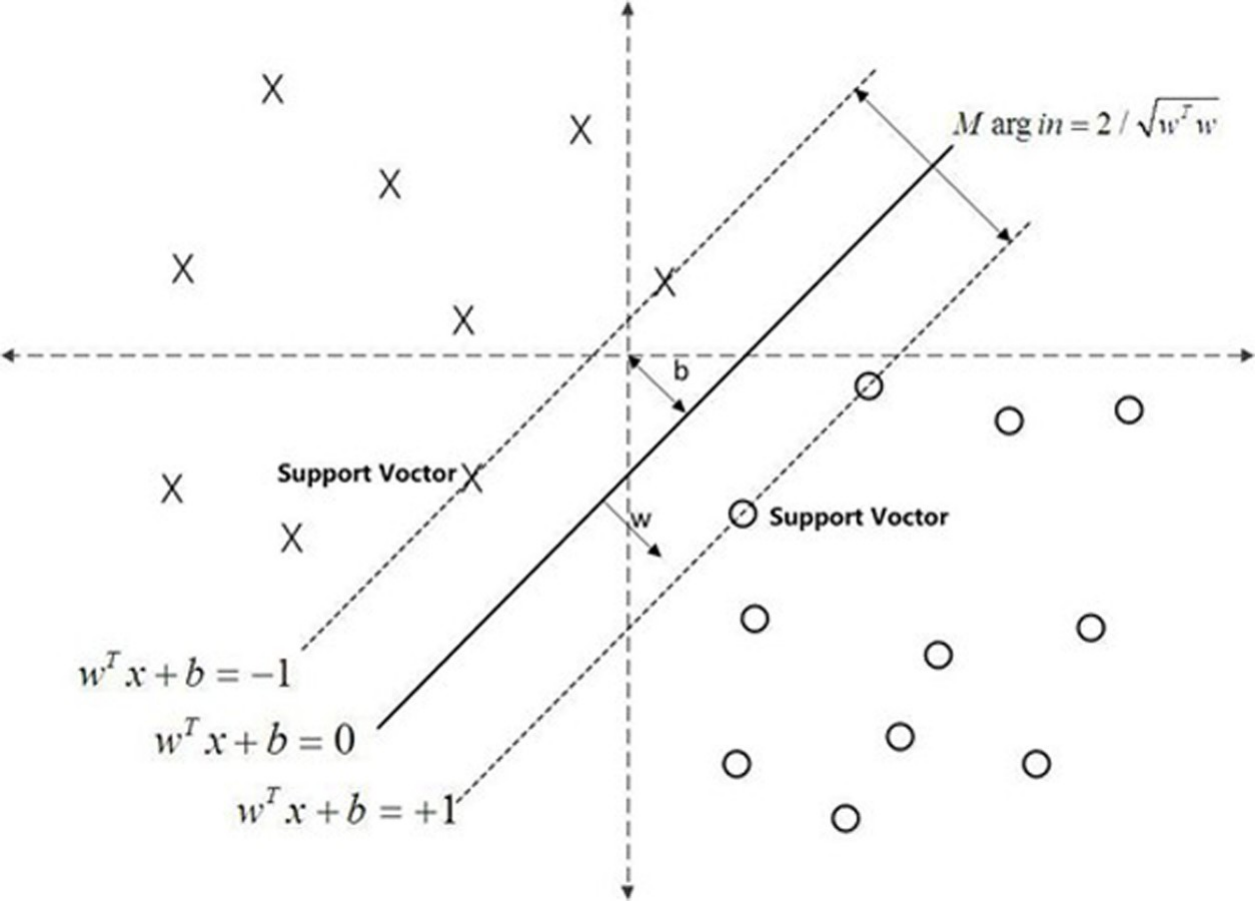
Support vector machine diagram.

A support vector machine (SVM) is a generalized linear classifier that performs the binary classification of data in a supervised learning way, which is mainly used to solve the problem of how to classify samples. The model is not demanding in terms of data volume and thus is widely used in prediction studies. The SVM model is applied to the prediction of loan acquisition. The method tries to find the hyperplane to separate the successful loan acquisition from the failed target. It is mainly through the transformation of the kernel function from low-dimensional space to high-dimensional space that linear divisibility is achieved. It can be further understood as a linear classifier with the largest spacing in a feature space, as shown in **Fig 2**. In the figure, the separation hyperplane is represented by a straight line, w^*T*^x+b = 0. The two dotted lines in the figure represent two hyperplanes at a specific distance from the solid line (set as 1 in the figure), and the support vector machine is the point on the two dotted lines.

## 3. Empirical study

### 3.1 Descriptive statistical analysis of variables

In this paper, descriptive statistical analysis is carried out on the linguistic feature variables and control variables of loan descriptions, and the results are shown in **Table 4**. It can be seen that in all samples, the lending success rate is 21%, indicating that the overall lending success rate is not high. The average loan amount is 62,463.61 CNY, and borrowers’ education is generally a junior college or bachelor’s degree. In terms of credit status, 71% of borrowers provide credit report certification. The average credit limit is 56,279.32 CNY.

**Table 4:** Description statistics of control variables and linguistic feature variables.

Variable	N	Min	Max	Mean	S.D.	Skewness	*Kurtosis*	Jarqe-Berra
**Dependent variable**								
Success	20349	0	1	0.21	0.40	1.45	0.11	14236.75^***^
**Control variables**								
Amount	20349	3000	480000	62463.62	37415.55	1.28	3.62	5875.98^***^
Educational level	20349	0	4	2.05	0.75	0.16	-0.62	11193.76^***^
Credit limit	20349	0	480000	56279.32	40381.52	1.34	4.67	8493.66^***^
Credit report	20349	0	1	0.71	0.45	-0.94	-1.12	17367.95^***^
Successful record	20349	1	32	1.08	0.51	3.89	6.20	60077.26^***^
**Linguistic feature variables**								
Length of sentence	20349	7	485	104.37	48.09	1.93	6.42	22534.20^***^
Number of valid characters	20349	4	331	76.98	35.49	1.95	7.02	26617.83^***^
The ratio of effective characters	20349	0.10	1	0.74	0.05	-2.92	16.24	177477.75^***^
Number of periods	20349	0	55	2.77	1.09	0.20	11.67	63798.73^***^
Ratio of complex words	20349	0	0.50	0.01	0.02	5.84	26.71	592354.24^***^

In the descriptive statistical analysis results of the linguistic feature variables of the loan description, the average length of the description text is 104.37 characters, and the average number of valid characters is 76.98. The average proportion of valid characters is 71%, the average number of periods is 2.77, and the average proportion of complex words is 0.01. The statistical results show that the descriptive text of the borrower is mainly composed of long sentences, and the borrower tends to provide a more complete description of the loan. All variables were skewed to the right, except for the Credit report and Ratio of effective characters, which are left-skewed. The Jarque–Bera test showed that all variables were non-normally distributed at the 1% level.

### 3.2 Correlation analysis and collinear diagnosis of control variables

Based on previous studies, this paper selects 7 variables to form an indicator system of the control variables. It is uncertain whether there is a significant correlation between these variables and the dependent variable, " Access to loans or not ". To make a preliminary judgment on the correlation between variables, bivariate correlation analysis should be carried out for each variable and dependent variable.

**Table 5** below shows the results of the Pearson correlation analysis. As seen from the table, all the control variables can show significance at a 1% level. Therefore, it can be considered that the selection of these seven variables has an influencing relationship with the dependent variables and that the control variable system constructed is effective.

**Table 5:** Results of Pearson correlation analysis.

	Amount	Educational level	Credit limit	Credit report	Successful record
Amount	1				
Educational level	0.094^***^	1			
Credit limit	0.268^***^	-0.006^***^	1		
Credit report	0.073^***^	0.079^***^	0.123^***^	1	
Successful record	-0.220^***^	0.015^***^	0-.087^***^	-0.047^***^	1

Note: *, **, and ***, respectively, denote significance at 10%, 5%, and 1%. The same as below.

In this paper, when exploring the influence of loan description information on loan acquisition, the regression model established is based on the logistic regression model. In the construction of this model, the effect of multicollinearity between explanatory variables on the accuracy of results should not be considered. Therefore, the multivariate collinearity diagnosis should be carried out first. For the diagnosis of multicollinearity, the commonly used reference statistics are variance inflation factor (VIF) and tolerance (TOL). When VIF<10, it indicates that there is no obvious multicollinearity relationship between variables. The tolerance is the reciprocal of VIF, namely TOL = 1/VIF, and the value range is (0,1). The closer the tolerance is to 0, the stronger the multicollinearity. The results of the multicollinearity diagnosis of the control variables are shown in **Table 6**. The results show that the VIF of all control variables is less than 10, and the tolerance is not close to 0. Thus, there is no multicollinearity problem with the control variables, and the control variable system is established to meet the requirements.

**Table 6:** Results of collinearity diagnostics.

Variable	Amount	Educational level	Credit limit	Credit report	Successful record
VIF	1.138	1.017	1.093	1.024	1.054
TOL	0.879	0.983	0.915	0.976	0.949
N	20349	20349	20349	20349	20349

### 3.3 Empirical analysis results

#### 3.3.1 Endogeneity test and regression analysis

This paper constructs regression models before and after the addition of variables related to loan description information to analyze whether the loan description has an impact on loan acquisition. **Table 7** shows the results of the regression analysis for models 1–4.

**Table 7:** Results of regression analysis of the effect of loan description on loan acquisition.

Variable	(1)		(2)		(3)		(4)	
	Logistic	OLS	Logistic	OLS	Logistic	OLS	Logistic	OLS
Control variables								
Amount(log)	-3.950^***^	-0.301^***^	-.778^**^	-0.125^***^	-3.820^***^	-0.290^***^	-0.792^**^	-0.117^***^
Educational level	-0.218^***^	0.003	-0.260^***^	0.003	-0.225^***^	0.003	-0.253	0.003
Credit limit(log)	-2.648^***^	-0.182^***^	-3.345^***^	-0.184^***^	-2.723^***^	-0.181^***^	-3.367^***^	-0.184^***^
Credit report	-1.278^***^	-0.071^***^	-1.237^***^	-0.060^***^	-1.252^***^	-0.069^***^	-1.217^***^	-0.059^***^
Successful record	1.164^***^	0.081^***^	0.805^***^	0.050^***^	1.162^***^	0.079^***^	0.809^***^	0.048^***^
Linguistic feature variables								
Length of sentence			0.115^***^	0.001			0.114^***^	0.001
Number of valid characters			-0.198^***^	-0.002^***^			-0.196^***^	-0.002^***^
The ratio of effective characters			2.684^**^	0.920^***^			2.661^**^	0.892^**^
Number of periods			-14.241^***^	-2.790^***^			-14.556^***^	-2.753^***^
Ratio of complex words			-11.225^***^	1.898^***^			11.6^***^	1.847^***^
Content features variables								
Theme 1					1.099^***^	0.040^***^	0.984^***^	0.029^***^
Theme 2					0.006	0.010	-0.427^**^	0.001
Theme 3					1.453^***^	0.063^***^	1.267^***^	0.051^***^
Theme 4					1.598^***^	0.080^***^	1.419^***^	0.067^***^
Constant	27.668	84.791	17.899	62.171	26.363	79.441	17.135	59.493
N	20349	20349	20349	20349	20349	20349	20349	20349

*(1) Discussion about the endogeneity issue*. Endogeneity issues can interfere with the accuracy of results [25]. The aim of this paper is to analyze the impact of loan descriptions on lending outcomes. A number of orders and publicly available information about the borrower were selected as control variables (amount, success records, etc.). It is difficult to avoid the association between these variables. That is, there is endogeneity.

However, the indicators of loan descriptions consist of linguistic and content features, which reflect the linguistic structure and content themes of the text, and there is no obvious connection between them and the control variables. It would also not significantly interfere with the purpose of the analysis. Therefore, we do not develop too much analysis of the endogeneity issue.

In addition, to ensure the accuracy of the regression results, this paper uses OLS regression along with logistic regression. The regression results are shown in Table 7. It can be seen that among the control variables, except for education, the coefficients of OLS and logistic regression remain consistent in terms of positivity, negativity and significance, which indicates that the regression results are reliable.

*(2) Regression analysis*. Column 1 shows the regression results of the original model (1), reflecting the impact of the seven control variables on loan acquisition. The data in the table show that all the variables are significant at the level of 1%. This indicates that there is a significant correlation between each control variable and the availability of loans.

Column 2 shows the regression results of model (2). After adding the linguistic feature variables of loan description based on control variables, the model conducts regression again. The purpose is to explore whether the linguistic features of loan description can influence loan acquisition. Table 7 shows that the more text characteristics that are described in borrowing and lending, and the higher the ratio of effective content, the higher the possibility of successful lending. The number of periods or clauses will reduce the likelihood to obtain a loan. In terms of significance, all variables are at the level of 1%, except the ratio of effective characters is 5%.

Regression model (3) is obtained after the characteristic variables representing the loan description are included based on the control variables. Except for theme 2 (loan purpose), all the other themes (working condition, asset, and image-building content) show significant relationships with borrowing success.

Column 4 is the result of the regression model (4), obtained after adding the relevant variables of the language and content features of the loan description (except theme 2) into the original model. Among the linguistic feature variables, the only ratio of effective characters for a significant proportion at the 5% level, other variables at the level of 1%. Among the content characteristic variables, all show significant performance at the 1% level.

#### 3.3.2 Regression results

The results show that in the loan description text, if the borrower explains his/her work condition and assets, then the probability of success of the loan can be improved, expressing that the purpose is not valid. Besides, the borrower can be easier to get a loan by building a good image of themselves. Such as expressing greetings, gratitude, repayment guarantee, and other content in the loan description. The regression results of model (4) show that the more complex the language structure of the loan description text, the more difficult the loan application will be. Regarding content, lenders are more concerned about the purpose of the borrower’s loan. When the borrower explains his/her work condition and assets, it can also improve the possibility of him/her obtaining this online loan.

According to the significant results, it is found that in the process of P2P online lending transactions, the description text of the loan carried out by the borrower can indeed have a significant impact on the judgment of lenders. In terms of content, text that can provide a detailed description of the borrower’s work and assets can improve his/her possibility of obtaining the loan in terms of the subprime loan application. In addition, the content written by the borrower to build a good image and increase lender goodwill, such as ‘Thank you, I promise to pay you back regularly’, and ‘Honesty is the most important, some loans and some repayments’, will significantly improve the success rate of the loan.

The empirical results show that the loan description can indeed have an impact on the success of online lending, the language and content dimensions can have a corresponding impact, and the content aspect has a more obvious impact. In terms of the language structure of the loan description text, it should be kept as concise as possible, as the frequent use of complex words will create obstacles for investors in reading, and thus increase the difficulty involved in obtaining this online loan. The loan description should focus on the borrowers’ economic asset status. In addition, some greeting and guarantee words in the loan description can improve the possibility of borrowing. It will increase the trust of investors, making it easier to obtain online loans. And Investors’ impression of the borrower can significantly affect the outcome of the loan.

### 3.4 Prediction of online lending results based on loan description

#### 3.4.1 Variable selection for prediction

After the establishment of the regression model and the empirical analysis, it can be proved that in the process of online lending transactions, the loan description has very important value for investors, who can obtain information on the tendency of the borrower by reading his/her loan description. If the loan description is written in a language that is easily readable by the investor and contains specific points about which the investor is concerned, then the loan application is more likely to be successful.

In terms of variable selection, the explanations of the variables used in the process of prediction analysis in this section are the same as those in the above influence factor analysis.

To explore the role of the loan description in the prediction of loan results, it is necessary to first determine the hard information-related variables as the original variable system of comparative analysis. This section of the original variable system is consistent with the analysis of Section 3.2, and the amount, interest, educational level, credit rating, credit limit, credit report, successful record variables are considering hard information in terms of forecast analysis index system, joining the description information feature variables from the borrowing prediction results.

In the selection of soft information variables such as descriptive information, variables that are significant at the 1% and 5% levels in the impact analysis of the lending results are mainly selected and include complex word proportion, Theme 1 (work condition), Theme 2 (loan purpose) and Theme 3 (asset).

To further explore the value of the loan description in the research on loan result prediction, the SPSS Modeler software was used to select the support vector machine (SVM model) as the classification prediction algorithm, and the prediction results before and after the loan description were added were compared and analyzed.

#### 3.4.2 Results of loan forecasting

This section divides the total data set according to the standard that the ratio between the training set and test set is 7:3. The training set, accounting for 70% of the total data set, is used to fit the model, and the remaining test set data, accounting for 30%, is used to evaluate the performance of the model. To better reflect the forecast results, after classification prediction, as the confusion matrix for specific prediction results shows, and based on the confusion matrix, the rate of accuracy (ACC), precision (PPV), true positive rate (TPR), true negative rate (TNR), F value and AUC value are selected to compare related indicators, and the ROC curves are more intuitive.

**Table 8** shows the obfuscation matrix obtained by using a support vector machine (SVM) to classify and predict the data set. Based on the confusion matrix, the value of each evaluation index is calculated, and **Table 9** is presented. In the two tables, (1) represents the prediction of the lending results by the support vector machine model when only the control variable system exists, and (2) represents the prediction results of the model after the relevant variables of the borrowing description are added.

**Table 8:** Confusion matrix.

	Training set			Test set		
(1)	Observed value	Predicted value		Observed value	Predicted value	
		1	0		1	0
	1	1581	1377	1	660	579
	0	361	10929	0	138	4724
(2)	Observed value	Predicted value		Observed value	Predicted value	
		1	0		1	0
	1	1721	1237	1	715	524
	0	254	11036	0	100	4762

**Table 9:** Evaluation index of the SVM.

Test set	ACC	PPV	TPR	TNR	F-score
(1)	0.8379	0.7615	0.3195	0.9738	0.4501
(2)	0.8606	0.7949	0.4429	0.9701	0.5688
Training set	ACC	PPV	TPR	TNR	F-score
(1)	0.8397	0.7554	0.3115	0.9743	0.4411
(2)	0.8669	0.8144	0.4463	0.9741	0.5766

According to the numerical situation in **Table 8**, after adding the relevant variables of loan description, the values of true positive (TP) and true negative (TN) in the confusion matrix of the training set and test set increase, while the values of false positive (FP) and false negative (FN) decrease, and the prediction accuracy of the corresponding model is improved. In **Table 9**, except for the small change in the true negative rate (TNR), the other indicators have been increased. In the evaluation index of the prediction model, accuracy (ACC) reflects the accuracy of the overall prediction of the model, while the true rate (TPR) represents the ratio of the actual positive samples to the predicted positive samples. Therefore, based on **Tables 8** and **9**, it is found that the prediction effect of the support vector machine model is significantly improved after the addition of loan description characteristic information. This part of the optimization is mainly achieved by improving the prediction accuracy of the positive samples.

**Fig 3:**
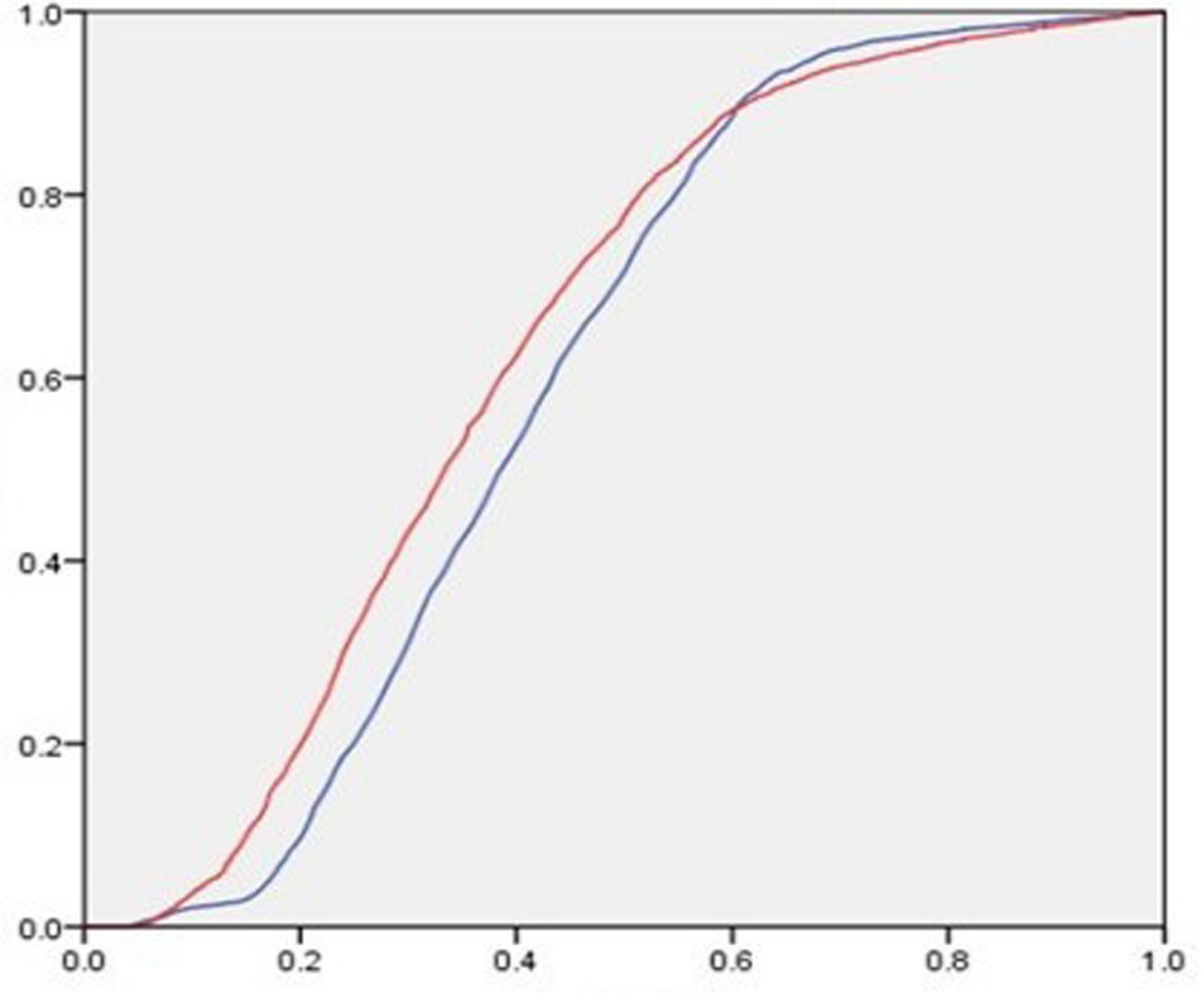
The ROC curve of the test set is predicted by SVM.

Based on the results of the confusion matrix, the ROC curve of support vector machine (SVM) prediction was drawn with the false positive rate (1-specificity) as the abscissa and the true rate (confidentiality) as the ordinate. By comparing the trends of the two curves before and after adding the loan description information, the performance changes of the model are compared. The blue line represents before and the red line represents after. The ROC curve is shown in **Fig 3**. The closer the graph is to the upper left corner, the higher the prediction accuracy of this model. It can be seen from the figure that the ROC curve reflects the same situation as that in **Tables 8** and **9**, and the loan description information can significantly improve the accuracy of the support vector machine model in predicting the loan results.

#### 3.4.3 Robustness test of forecasting results

To ensure the reliability of some of the abovementioned results, tests of results are needed. In this part, this paper attempts to verify that the loan description can play an optimized role in the loan prediction results by changing the proportion of the test set and replacing other classification prediction models.

In addition to the support vector machine (SVM) model, logistic regression, random forest (R-F), neural network (NNET) and other models have relatively stable performance and better predictive stability among many data mining algorithms applied to the research on prediction of network lending results. Therefore, this section will choose these four classifier models to compare and analyze the performance of each model before and after adding the loan description, to verify the predictive optimization function of the loan description information. At the same time, the ratio of the training set to the test set was changed to 8:2 and 6:4, respectively, and the experiment was carried out separately and compared with the result at 7:3.

**Fig 4:**
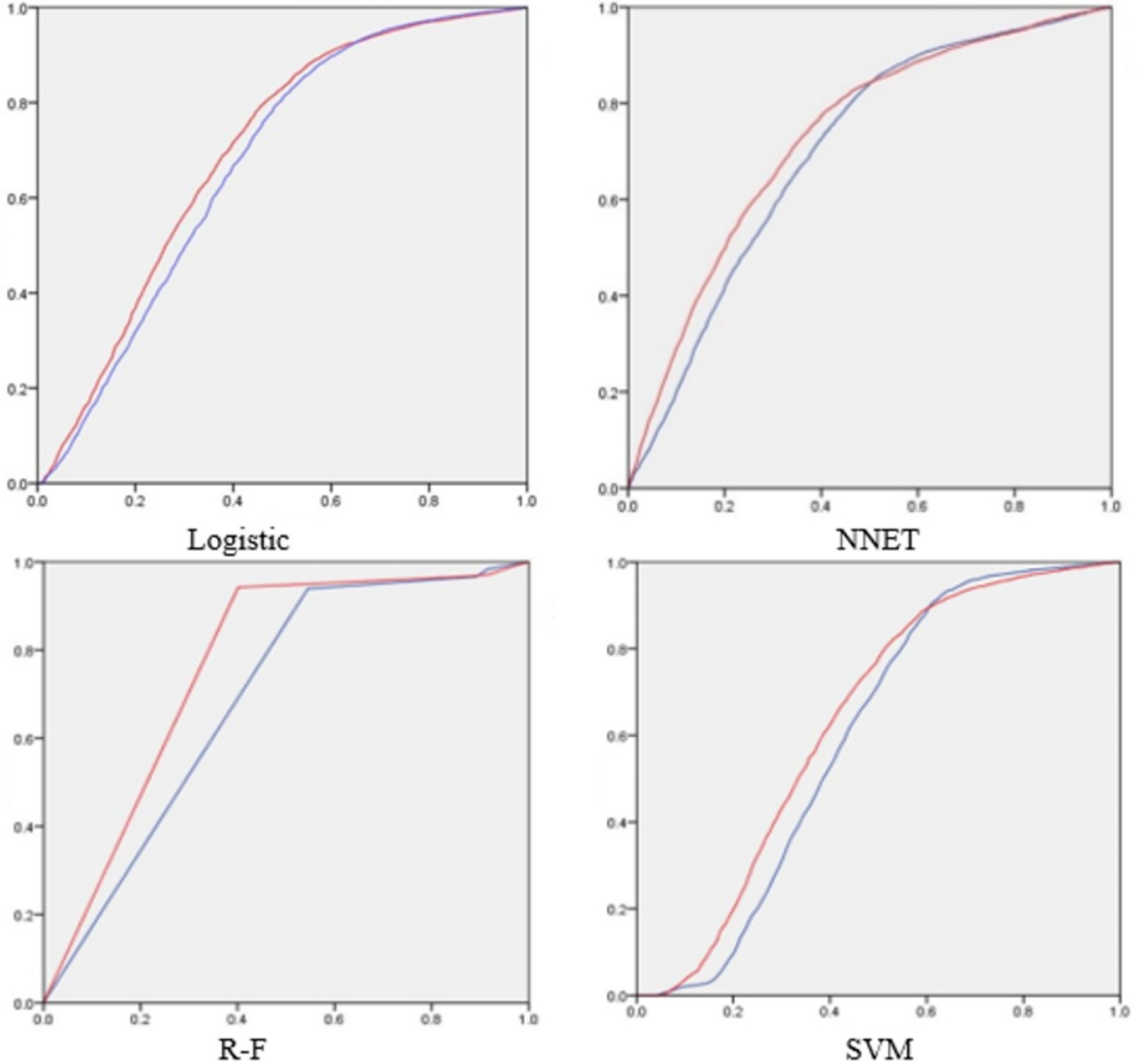
ROC curve changes of each model.

**Table 10** shows the confounding matrices of the four models in the case of 80% and 60% training set ratios. Because the training set and the test set are similar in numerical performance characteristics, only the specific situation of the training set is given. Regardless of the prediction model, when the loan description-related variables are added, the TP value in the confusion matrix is increased and the FN value is decreased. It is verified that the loan description information can improve the prediction accuracy of the model for the positive sample and reduce the prediction error for the negative sample. **Fig 4** shows the impact of the addition of loan description information on the ROC curves of the four models. The situation in the figure shows that regardless of the type of forecasting model, the loan description information can improve the prediction accuracy of the model.

**Table 10:** Confusion matrix of each model with different proportions of the training set.

Training set accounts for 80%		TP	FN	FP	TN
Logistic	(1)	1063	2293	355	12571
	(2)	1351	2005	376	12550
NNET	(1)	1297	2059	537	12389
	(2)	1618	1738	448	12478
R-F	(1)	1445	1911	569	12357
	(2)	1712	1644	375	12551
SVM	(1)	1056	2300	343	12583
	(2)	1467	1889	380	12546
Training set accounts for 60%		TP	FN	FP	TN
Logistic	(1)	836	1711	260	9366
	(2)	1062	1485	268	9358
NNET	(1)	1016	1531	396	9230
	(2)	1265	1282	324	9302
R-F	(1)	1115	1432	418	9208
	(2)	1331	1216	278	9348
SVM	(1)	827	1720	247	9379
	(2)	1151	1396	282	9344

## 4. Conclusions

The loan description is a subjective reflection of borrower information in P2P online lending and an important basis for investors to establish trust in the transaction process, and it can alleviate information asymmetry between lenders and borrowers to a certain extent. This paper takes the data of ‘Renrendai’, one of the representative platforms of the P2P industry in China, as the research sample to explore the value of the loan description in the process of online lending transactions. First, the loan description text in the sample data is quantified and analyzed, the relevant indexes of the loan description are constructed from two characteristic dimensions of linguistic features and content features, and the influence of the loan description text on the acquisition of online loans is explored through the establishment of regression models.

The empirical results show that factors such as invalid characters, complex words, and sentence composition in the loan description text significantly impacted the success of borrowing. In terms of content, lenders pay more attention to the job and property status of borrowers. Empirical results show that, the success of the loan is influenced by whether the borrower’s own employment and assets are clearly discussed in the loan description. Although many borrowers stated the purpose and purpose of the loan, these did not seem to be taken into account by lenders. In addition, the portrayal of the borrower’s image through loan description is also an important basis for the lender to make a judgment. In the empirical study, it is verified that the repayment guarantees and commitment affects the lender’s willingness to invest.

The loan description information also has some value in optimizing the prediction of the loan results. After adding the variables related to the loan information into the support vector machine model, the prediction results of the model have been significantly improved. The prediction accuracy of the positive sample is improved, and the error prediction of the negative sample is reduced. The overall improvement is more than 3 percentage points, especially the accuracy of TPR, which has improved to about 10 percentage points. This conclusion remains robust under different data sets and different classification prediction models (logistic, neural network, and random forest).
